# Exploring clinical trials awareness, information access and participation amongst Australians with ovarian cancer: a qualitative study

**DOI:** 10.1007/s00520-025-09221-2

**Published:** 2025-02-11

**Authors:** Natalie Williams, Hayley Russell, Bridget Bradhurst

**Affiliations:** 1 Ovarian Cancer Australia, Melbourne, VIC 3000 Australia; 2 Curtin University, Bentley, WA 6102 Australia

**Keywords:** Ovarian cancer, Clinical trials, Qualitative research, Awareness, Gynaecological cancers, Ovarian neoplasms

## Abstract

**Purpose:**

Ovarian cancer is associated with advanced stage diagnosis and poor survival rates. Clinical trials are critical for improving both clinical and quality of life outcomes. Challenges exist to clinical trials awareness, information access and participation, but perspectives of Australians with ovarian cancer have not been previously investigated. We aimed to explore clinical trials awareness, information access and participation amongst Australians with ovarian cancer.

**Methods:**

Utilising an exploratory qualitative approach, women with ovarian cancer participated in online focus groups and interviews between December 2023 and February 2024. Transcripts underwent inductive content analysis.

**Results:**

Five themes and five subthemes emerged. In theme 1, participants identified “Barriers exist that affect clinical trial awareness and participation” and were explored through their experience of ovarian cancer. In theme 2, participants shared that “Instigating the conversation and doing my own research” was necessary to access clinical trials. Theme 3 describes ideas on “Finding solutions to improve clinical trial awareness and information access” through subthemes: “we need a centralised, credible source”; “communicate clinical trials in various ways from trusted contacts”; and “I want tailored, relevant information”. Theme 4 explained that “Altruism is a motivator” in willingness to participate in trials. Finally, in theme 5, participants explained that “Emotions regarding clinical trials are varied” illustrated in subthemes: “feeling left behind” and “feeling fortunate”.

**Conclusions:**

These qualitative insights will inform development of a cross-sectional survey for national distribution amongst Australians with ovarian cancer. Results will assist in developing solutions to improve clinical trials awareness and information access.

**Supplementary Information:**

The online version contains supplementary material available at 10.1007/s00520-025-09221-2.

## Introduction

Ovarian cancer is a disease commonly associated with advanced stage diagnosis and poor survival [1, 2]. Due to their similarities, cancers arising in the fallopian tube and peritoneum are also commonly included under the ovarian cancer designation [3]. Worldwide, ovarian cancer is diagnosed in over 300,00 people each year [4], and latest estimates suggest over 5000 Australians are currently living with this disease [1]. Ovarian cancer affects organs exclusive to the female reproductive system. In this paper, people affected by ovarian cancer are at times referred to as “women”, and in the pursuit of inclusivity, the authors wish to recognise there are gender diverse people affected by ovarian cancer [5] who may not identify with this gendered term.

Clinical trials are a critical component of efforts to improve clinical and quality of life outcomes in ovarian cancer care aiming to benefit both people diagnosed in the future and those currently living with the disease. Clinical trials may be used to test new treatments, therapeutic interventions and supportive care services through all time points of the cancer experience from diagnosis to survivorship. Importantly, in ovarian cancer where recurrence is common, clinical trials can also present an opportunity to receive crucial alternative treatment options when initial efforts have been exhausted [6].

Challenges exist to clinical trials awareness, information access and participation. A Cochrane review investigating factors related to trial recruitment in healthcare found the way the trial is set up and communicated, personal circumstances and possible benefits influenced decision to join the trial [7]. A systematic review including studies exclusively from the USA suggests trial participation is unachievable for the majority of cancer patients, and there is a significant need to address clinical and structural barriers [8]. Also in the USA, discordant attitudes about cancer trial participation between clinicians and patients are recognised, and it is recommended that improving communication and addressing infrastructure and resourcing issues is needed to increase recruitment [9].

In Australia, investigation of barriers and enablers to clinical trial recruitment found the most common factor influencing 162 consumers’ decision to participate was awareness of a relevant trial (88.0%) [10]. Awareness of clinical trials is not commonly reported in published literature. A recent cross-sectional survey of 242 Australians with ovarian cancer asked if participants had been told about or offered to take part in a clinical trial [11]. A marginal increase of respondents being asked about clinical trials was reported over an 8-year period (65.2% in 2015 to 69.8% in 2022) [11]; however, no further exploration of factors influencing awareness and how this affects the lived experience of ovarian cancer was undertaken.

A non-government, not-for-profit organisation providing support and advocacy for people affected by ovarian cancer in Australia (Ovarian Cancer Australia) is responding to community feedback to progress resource development for improved access to clinical trials information and participation [12]. This approach is supported by international consensus that states cancer care should focus on outcomes that matter to patients [13]. Concurrently, the examination of barriers and enablers influencing cancer trial participation more broadly across all tumour types is underway in Australia; however, this proposed Delphi study seeks to investigate stakeholder perspectives and does not include people with a lived experienced of cancer [14]. Acknowledging the role clinical trials play in ovarian cancer care and to address this gap in literature, our study aimed to explore the perspectives of Australians with ovarian cancer on clinical trials awareness, information access or participation.

## Methods

An exploratory qualitative descriptive approach was chosen to explore participants’ experiences, an approach used commonly in health research which recognises varied shared experiences and summarising common ideas [15]. Pragmatism underpinned this approach where collaboration with people with lived experience was key to exploring the study concept and developing solution ideas [16]. The Consolidated Criteria for Reporting Qualitative research (COREQ) checklist was used to guide the presentation of the results throughout this paper ensuring comprehensive and transparent reporting [17] and is provided as a supplementary file.

### Participants and recruitment

To meet the study’s inclusion criteria, participants were required to have experienced a diagnosis or recurrence of ovarian, fallopian tube or peritoneal cancer within the previous 3 years, live in the state of New South Wales (NSW), Australia, be at least 18 years of age and proficient in reading and speaking English. All others were excluded.

Participants were recruited by convenience sampling using a targeted population. To explore the subject of interest amongst the population with lived experience and for whom solutions to identified barriers would benefit, an email invitation to participate in an online focus group or interview was distributed to people on the Ovarian Cancer Australia database. This database allowed for filtering of participants meeting the eligibility criteria and members had provided permission to be contacted by the organisation leading the research. Twenty-five respondents expressed interest in participating via an online survey on the Curtin University REDCap survey platform [18]. A second email invitation was distributed 8 weeks after the initial invitation highlighting expanded inclusion criteria to comprise those experiencing a recurrence of disease within the past 3 years resulting in a further seven respondents.

In total, 21 respondents provided written consent to participate. Four people were subsequently unable to attend an online focus group or interview and one was deemed ineligible, resulting in a total of 16 participants. This met the estimated sample size of between 12 and 20 participants, informed by the population size and considered appropriate in qualitative research [19]. Participants were allocated to focus groups based on their availability at the range of time options offered. If unable to attend available options, participants were offered an individual interview at a time convenient to them.

### Data collection

Basic demographic information including age, gender, locality classification, self-identified ethnicity and Aboriginal or Torres Strait Islander status were collected using an online survey. Eleven participants joined one of three online focus groups and five completed an online one-on-one video interview using the Microsoft Teams platform at a location of their choosing. Online focus groups and interviews were conducted between December 2023 and February 2024. Focus groups averaged 67 min and interviews 34 min in length. In total, 6 h and 12 min of verbal data was collected.

The primary author (NW) facilitated all focus groups/interviews and one other author (HR, BB) attended each focus group. The primary author is a female nurse researcher with 8 years of research experience, has completed a Master of Philosophy and was working as a researcher and program manager at the time of data collection. Participants were previously unknown to the facilitator. The facilitator’s experience, credentials and reasons for conducting the research were introduced to participants prior to focus group/interview commencement.

Focus groups/interviews were facilitated using a semi-structured guide (Table 1) developed by the authors using principles described by Liamputtong [20] to facilitate dialogue addressing the aims of the study. Verbal consent was confirmed at the commencement of each focus group/interview. Focus groups/interviews were recorded with permission and transcribed using automated transcription in Microsoft Teams. Transcripts were revised and edited after completion and references to names were removed to maintain anonymity during analysis. Transcripts were not returned to participants for correction, and no interviews were repeated. Field notes were completed after each focus group/interview. After the second round of recruitment and the inclusion of 16 participants, data saturation was reached. This was determined as no additional codes were being generated after analysis of the final two interview transcripts [21].

**Table 1:** Interview guide

**Introductory question**
Please share your understanding of what clinical trials are and how they aim to benefit people with cancer.
**Transition questions**
Have any of your treating team discussed access to clinical trials with you? If yes, how did they approach this? If no, why do you think they didn’t? Have you participated, or are you currently participating in a clinical trial? If yes, how did you find out about it? If no, why not?
**Focus questions**
What are the things you feel would help women with ovarian cancer to find out about clinical trials? What factors do you feel would help women with ovarian cancer to be referred to or enrol in clinical trials? What factors do you feel are barriers to women with ovarian cancer finding out about clinical trials? What factors do you feel are barriers to women with ovarian cancer enrolling in clinical trials? How would you like to receive information about clinical trials? What resources would help you or other women with ovarian cancer to find out about clinical trials?
**Summarising question**
We’ve been discussing your experiences of finding out about clinical trials for people with ovarian cancer. If you think back over our conversation today, can you describe the top three things you feel would help women with ovarian cancer to find out more about clinical trials?
**Concluding question**
Is there anything else about ovarian cancer clinical trials that we have missed or that you would like to share today?

### Data analysis

**Fig. 1:**
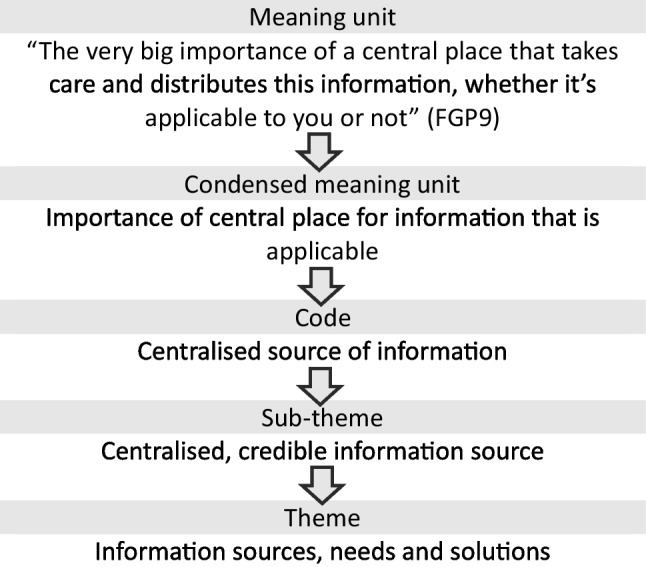
Sample of inductive content analysis following steps outlined by Erlingsson and Brysiewicz [23]

A qualitative conventional content analysis described by Hseih and Shannon [22] was conducted to summarise findings into key concepts based on the study aim. Conventional content analysis is considered appropriate when the aim is to describe a phenomenon where there is limited existing evidence of the concept being explored. Data analysis used a reflexive, inductive process, following the steps described by Erlingsson and Brysiewicz [23]. These included (1) data familiarisation by reading and re-reading transcripts; (2) dividing text into meaning units describing one idea per unit; (3) condensing meaning units in fewer words whilst keeping the central meaning intact; (4) formulating codes by applying descriptive labels to meaning units; (5) developing categories by grouping codes into the who, what when and where; and (6) developing themes by expressing the underlying meaning and addressing the ‘why, how, in what way and by what means’. Figure 1 shows a sample of this process.

The primary author conducted initial coding on all transcripts using NVivo software [24]. A second author (HR) undertook simultaneous coding of one focus group to ascertain reliability. All data from focus groups and interviews were analysed collectively. After initial coding, all authors met to discuss categorisation of codes and theme development. A grid of codes, categories, themes and supporting quotes was developed to ensure comprehensive and systematic inclusion of all data in the final results. Findings were not returned to participants for comment but were presented verbally to two people meeting the eligibility criteria who confirmed them as resonating with their own experience.

Demographic data were analysed using descriptive statistics in Microsoft Excel.

### Ethical considerations

This study was conducted in accordance with Australian standards for the ethical conduct of research [25, 26], jurisdictional guidance for implementing the Declaration of Helsinki [27]. The Curtin University Human Research Ethics Committee reviewed and approved the study (HRE2023-0637).

## Results

### Participant characteristics

Sixteen Australians participated either in focus groups (*n* = 11) or interviews (*n* = 5). Participant characteristics are provided in Table 2.

**Table 2:** Participant characteristics

	*n* (%, OR range)
**Mean age in years**	57 (44–74)
**Data collection method**	
Focus group Interview	11 (69) 5 (31)
**Time since diagnosis**	
Within 3 years	16 (100)
**Australian state/territory of residence**	
New South Wales	16 (100)
**Location of residence**	
Major metropolitan city Large regional town Small regional town Rural or remote location	11 (69) 1 (6) 3 (19) 1 (6)
**Self-identified gender**	
Female	16 (100)
**Self-identified ethnicity**	
Australian or mixed Australian	16 (100)
**Aboriginal and/or Torres Strait Islander**	
No	27 (100)

### Themes and subthemes

Five themes and five subthemes emerged from the data (Table 3). In this section, themes and subthemes are presented with supporting quotations from participants. A coding system after each quotation (e.g. FGP13 or IP4) indicates which participants were in a focus group (FG) or interview (I). Additional quotations are also provided in Table 3.

- Theme 1: Barriers exist that affect clinical trials awareness and participation

**Table 3:** Summary of themes and subthemes along with additional participant quotations

Theme	Subtheme	Quotation
Barriers exist that affect clinical trials awareness and participation		“I’ve constantly asked about clinical trials and I’ve just found that it’s been very difficult to get the relevant information or get the right detail.” (FGP7) “Like if I had to go to Canberra or Sydney, compared to someone that lived in Sydney, [what] would it mean for me to, at my own cost, to travel down there and to stay in motels and things. That would make it a little bit harder for people who do have to travel as well.” (IP4) “It’s a rarer cancer…so it sort of eliminates me with some of the trials” (IP3) “Understanding the trials, it’s bigger than Ben Hur.” (IP4)
Instigating the conversation and doing my own research		“Just keep on pushing and asking. That… seemed to be about the best way to get some answers.” (FGP12) “I think it depends on where you’re being treated… and how much of an advocate you are for yourself in chasing this stuff up.” (FGP8) “I’ve said several times to my oncologist that I would be willing to be available for a clinical trial.” (FGP10) “I did see a… oncologist…while she was good she just she said to me… 'you’ve gotta keep one step ahead.'” (IP3)
Finding solutions to improve clinical trial awareness and information access	We need a centralised, credible information source	“I hope that out of this, that is what will come out of it, that there is this opportunity for a common area that everyone can go to to access cancer, you know the types of trials that would be available and that, you know, people like me have had access to.” (FGP8) “If the oncologists just said, ‘Ohh, you know, would you mind if we had someone contact you?’ Then flicked your name off to the ovarian cancer people or the breast cancer people and you just got one call and then you'd work out do you wanna keep that connection going or not, that could be a game changer.” (IP4)
	Communicate clinical trials in various ways from trusted contacts	“Rather than sifting through so many trials and trying to work out what’s it all about, just having someone advocating on your behalf… to say actually this person would be eligible for the trial, how can we… work it out so that we can get a spot for this?” (IP3) “Even if it was something that you had… when you sign up for your Teal nurse, so whether that was one of the questions that you were asked…I think it would be good to centralise it there.” (FGP12) “I was just thinking about the kit that they give you when you’re first diagnosed with cancer… you get this big package from the Cancer Council … maybe a leaflet in that.” (FGP1)
	I want tailored, relevant information	“Initially I was like ‘oh this is some complete cowboy kind of trial’… I started looking into my trial and realising actually this is almost standard practice in America and Europe… educating people that it’s not quite cowboy.” (FGP2) “It needs to be good information as well as to what each trial is about and how people do qualify and where they need to go.” (IP4)
Altruism is a motivator		“I think often we have to be very sick before we can go into a trial, so it may not benefit us, but it will help people in the future, so I’m willing to go into trials.” (FGP1) “I’m very happy to help anybody else.” (FGP5) “Even if it’s not necessarily for the benefit of you, what if you benefit from them down the track.” (FGP9)
Emotions regarding clinical trials are varied	Feeling left behind	“Whilst ovarian cancers are very, very deadly, it’s not as big, or prolific as say your… and I don’t want to take anything away from breast cancer, but I’ll use that as an example.” (IP4) “There are no trials for what I’ve got at the moment, and if they are, they’re in two hospitals that are fairly far away from me, you know, it’s a shame…. Because I'm happy to go on to more. I’ve got three dogs. I’m not gonna leave my dogs.” (FGP1) “Another thing which I find a bit frustrating is it’s sort of like your life is on the balance just in terms of… is there a spot… available on a trial. So it just seems a shame that because this particular trial that may be beneficial for me, there’s no spots available.” (IP3)
	Feeling fortunate	“Like even over the 10 years that I’ve nearly had it for it’s just gone in leaps and bounds, really. In what's coming up and I feel like soon we might even have a test for it. It’s all very positive in my mind.” (FGP12) I think I’m quite fortunate in that my medical team are pretty much across it [clinical trials].” (FGP8)

In discussing clinical trials for ovarian cancer, women shared barriers, either actual or perceived, regarding becoming aware of clinical trials and accessing information. Some barriers relating to non-modifiable, individual factors including older age, socioeconomic disadvantage and speaking languages other than English were identified. One woman reflected, “There’s a whole lot of women who are from disadvantaged backgrounds or non-English speaking backgrounds” (IP1).

Practical challenges were shared including access to a specialist ovarian cancer healthcare professional, reliable internet connectivity, ability to travel to a trial centre and having money to cover trial participation costs.

Some participants described diagnosis and treatment factors that negatively affected their ability to find out about or participate in clinical trials. These included their diagnosed ovarian cancer sub-type, and treatment-related side effects, such as having a blurry mind or fatigue. “Barriers for me would be… just the energy to invest in looking for information whilst I’m undergoing active treatment” (IP1).

Communication challenges were shared, such as healthcare professionals not offering information about clinical trials, being too busy, the lack of clinician awareness of suitable trials and inappropriate timing of information delivery. “They’re [the oncologist] pretty under the pump… they probably don’t have time to look up all that information… they’re probably bombarded” (IP5).

Knowing where to find information was perceived as a challenge when women experienced difficulty navigating information from the wide variety of sources available. “You’re trying to find information from all different sources” (IP3).

Participants’ ineligibility to join trials and a lack of availability of trials were recognised as barriers. “I just wasn’t offered anything because I didn’t qualify or there wasn’t a need” (IP4). Finally, women explained that confusing and difficult to understand information along with the use of medical terminology made it difficult to understand their options. “A lot of the time I don’t understand the lingo” (FGP6).

- Theme 2: Instigating the conversation and doing my own research

Women reflected on their need to self-advocate for information access using a variety of strategies. “I feel like I do need to do my own research and be my own strongest advocate” (FGP7). Participants described needing to instigate a conversation with health professionals to discuss clinical trials and asking questions to find out more. In some cases, clinicians had not discussed clinical trials unless women brought it up first. “I’m always the one that sort of brings it up and says, is there anything?… it’s me instigating that… question” (IP3).

Others described using their own search skills to find information online to relate back to their treating team for advice. “I started just researching myself, overseas trials. When I come back to my oncologist I've said, ‘What about this trial? What about this test?’” (FGP6).

- Theme 3: Finding solutions to improve clinical trial awareness and information access

While reflecting on barriers faced, along with where they accessed information and needing to do their own research, participants shared suggested solutions to improve clinical trials awareness, information access and participation.

A wide variety of information sources were accessed including online sources such as cancer or clinical trial organisation and registry websites, social media and video sharing platforms and online support groups. Other sources were health professionals (doctors, nurses and allied health professionals), other people with ovarian cancer, family and friends, and alternative health providers.

Three subthemes summarise suggested solutions to addressing identified barriers and improving information access and delivery.

- Subtheme: We need a centralised, credible information source

Participants frequently described wanting one central and trusted source to seek information about clinical trials. “I think that centralised repository… would be the best way to go forward” (FGP7). Credibility of information was highlighted as an important component and some suggested inclusion of a filter feature to optimise search relevance. It was acknowledged an Australian online database exists but even with filters, it was challenging to navigate, “I did the filtering and there’s 52 options to go through and six pages” (FGP2). In contrast, others did not want to filter but wanted to access all the available information to determine if it was relevant to them.

Participants suggested the option to contribute information to a centralised source in order to share it with others. One woman suggested she would like an advocate to help her find a trial. Another explained she wanted her “oncologist to help me rather than me doing all the research. They’re the experts. Why should I do all the research?” (FGP6).

- Subtheme: Communicate clinical trials in various ways from trusted contacts

Participants shared that while it was important to have one central place to access clinical trials information, they also wanted to communicate with trusted contacts about clinical trials in various ways and formats.

Individualised verbal communication was highlighted as one preferred communication mode, particularly when provided by professionals who patients are already in contact with, such as nurses, doctors (including GPs), trial coordinators and other advocates. One woman explained “I did have a Teal [ovarian cancer telehealth] nurse for a while…I didn’t even realise that she may have been an access point for information about clinical trials” (FGP10).

Information provided in a variety of written formats both hard copy and online was also recommended. Suggestions included email, text message, posters, brochures and trusted websites. “Emails are good. Yep, emails, text anything like that” (IP5).

Information accessibility was an important consideration, and some participants suggested wanting information delivered directly to them, rather than needing to look themselves. They wanted information delivery through health professionals within the treating team, automatic linkage of trial coordinators to participants or having an advocate to match people with trials they are eligible for.

- Subtheme: I want tailored, relevant information

Participants wanted information about clinical trials that was tailored and relevant to them as a person with ovarian cancer. As a starting point, participants suggested being provided with a basic understanding of what clinical trials are from which to then find out more information. Other topics they wanted information on included rights and risks of clinical trials including safety, how to withdraw from a trial and psychological impacts of participation. Participants wanted trial specific information, such as participant requirements, understanding the research question and trial progress updates. Finally, participants wanted to understand the impact of participation on improving care for others with ovarian cancer. “I’d wanna know where that [clinical trial] fits in… with what’s already available to people… clear understanding of what the benefits were” (IP1).

- Theme 4: Altruism is a motivator

When discussing clinical trials, participants reflected on their motivations to access information, stay informed and participate. Apart from finding a treatment to improve their prognosis, women expressed their perspectives that willingness to participate was linked to a sense of altruism and doing good for others. One woman said, “I’m gonna do my best for everyone out there, to help” (IP4).

Linked to this notion of altruism was participants’ desire to make a difference and to improve the care for future people with ovarian cancer. “I don’t mind if… it won’t help me, obviously I’d love it to help me, but if it it’s gonna help other people down the track, obviously that’s a bonus too” (FGP8).

- Theme 5: Emotions regarding clinical trials are varied

In describing their understanding and experiences of clinical trials awareness, women expressed a variety of contrasting emotions. These ranged from feeling positive about the future, living in hope and feeling fortunate, to fear, disappointment and frustration. These emotional perspectives are described below in two subthemes.

- Subtheme: Feeling left behind

Women shared how living with ovarian cancer and limited opportunities to participate in trials made them feel left behind other cancer types. They compared treatment, funding and research advances made in other tumour types, in particular breast cancer. “Ovarian cancer definitely needs money, and I always get a little bit titchy when breast cancer gets so much. I keep saying, what about me? What about ovarian cancer?” (FGP5). Some women with rarer sub-types of ovarian cancer shared there were fewer clinical trial options available to them compared with more common types.

- Subtheme: Feeling fortunate

Some participants connected their ability to access clinical trials information with feeling fortunate or lucky and suggested not everyone has the same access to information. One woman explained, “I feel very lucky that my surgeon worked with the surgeon running the clinical trial. I don’t know how that’s disseminated otherwise… You know, is that just luck of the draw?” (FGP2).

Another participant reflected on feeling fortunate to have attributes that made it easier for her to access clinical trial information, such as “I’ve got English as a primary language” (IP1) and “I work in health…I’m literate” (IP1).

## Discussion

This exploration of clinical trials awareness, information access and participation amongst 16 Australian women with ovarian cancer is the first of its kind in this population subset. In Australia, opportunities to participate in clinical trials may be limited by their availability in a relatively small disease population. In a disease where survival rates are low, improving clinical trials awareness and participation had been highlighted in community feedback as an area of need [12]. One of the primary goals of the organisation leading the research is to advocate for the needs of Australians with ovarian cancer; therefore, exploring their perspectives to support the development of resources to improve awareness was essential.

Findings from this study demonstrated that participants had an appetite for more information on clinical trials. They identified barriers faced in accessing information and participating in clinical trials and provided suggested solutions. Participants provided unique insights through the lens of ovarian cancer lived experience, contextualising their reflections by describing motivators and emotional impacts.

Best practice guidance in Australia states women with ovarian cancer have the right to access information about clinical trials suitable for them [28]. This echoes international consensus from the Common Sense Oncology Movement which declares patients should receive clear communication regarding treatment options [13]. Additionally, the World Ovarian Cancer Coalition Every Woman Study suggests a need to improve care for people with ovarian cancer including access to good quality information relevant to their disease [29].

Within international literature, several studies have investigated cancer clinical trial awareness; however, none explored this concept amongst participants with ovarian cancer. One qualitative study from the USA compared clinical trial awareness between inner-city and rural cancer patients [30]. The findings revealed both a lack of awareness and understanding about clinical trials along with misconceptions about what is involved. Similar to our study, healthcare providers were considered a crucial information source, particularly through verbal communication methods and approximately half of the 66 participants reported using online information sources [30].

Communication about and access to information about clinical trials is a concept recognised as challenging for potential trial participants. In the USA, studies of clinical trial websites and other recruitment resources found written information were beyond the average literacy levels of the population [31, 32]. In the cancer population, cognitive absorption is a recognised challenge for people undergoing treatment [33], including those with ovarian cancer [34, 35]. The impact of these challenges were similarly highlighted by participants in our study. Most of the existing information sources participants referred to were online, with descriptions of variable ease of access and with a level of detail not conducive to patient navigation. It was highlighted that many of these existing sources also rely on internet access, English proficiency and health literacy.

A repeated suggestion in response to challenges identified in our study was the concept of a centralised information source. Some participants explained that whilst they were aware of the existence of information in the form of publicly available online databases or registries, these existing sources are not meeting their needs. They explained existing sources can be challenging to navigate, with difficulty finding relevant information in language that is easy to understand, recognising again the challenge of comprehending complex clinical information when cognitive function has been impacted by treatment.

Assessment of clinical trials awareness amongst cancer populations has previously been investigated in Saudi Arabia [36], Korea [37], Poland [38] and the USA [39] using investigator-developed survey tools. No representation of people with ovarian cancer was reported within these studies. An investigation of the complementary concepts of knowledge, attitudes and trust in clinical research amongst women with ovarian cancer in Italy has been reported [40]. Recommendations include population-wide action to raise awareness of clinical trials amongst both women with ovarian cancer and those who are healthy to facilitate participation and decision-making.

Cross-sectional surveys are a suitable tool for measuring health-related attitudes and knowledge [41]. This method will constitute an important next step to build on the investigation of clinical trial information needs amongst Australians with ovarian cancer before investing in solutions to meet their needs. Previous studies of cancer clinical trials awareness and participation provide little detail on how their survey tools were developed and no process of validation was described [36–39]. In the absence of a validated tool, it is recommended the development of a survey tool should consider existing qualitative data [42], which did not exist for the Australian ovarian cancer population prior to our study.

In recognising that cancer care should focus on outcomes that matter to patients [13], exploring the perspectives of people with lived experience of cancer is critical in all aspects of care. Participants in our qualitative study demonstrated their motivation to participate in clinical trials for both their own benefit and that of others within the context of the emotional impacts of their cancer experience. Previous work from the USA [43] demonstrates the willingness of gynecologic cancer patients to participate in clinical trials, and improving patient awareness is recommended to help increase participation. Similarly, other Australian women with advanced gynaecological cancers view research as a vehicle for change and express their motivation for research participation as a service for knowledge production [44]. Partnering with consumers is critical when exploring challenges to clinical trials awareness and information access to ensure plans for newly developed resources adequately meet their needs. Our study provides this important foundation prior to the next steps outlined below.

### Future implications

This study forms part of a larger exploratory mixed-methods study investigating the perspectives of Australians with ovarian cancer on clinical trials awareness, information access and participation with the goal of developing resources to address barriers to information access. Despite consumer evidence to support the link between clinical trial awareness being a common factor in the decision to participate [10], no validated survey tools could be found to investigate these issues. The qualitative insights from this study provide crucial context and data to build on existing evidence and support the development of a survey tool to further investigate these issues on a wider scale.

### Strengths and limitations

A strength of this qualitative study was the exploration of a phenomenon amongst Australians with ovarian cancer that was not previously reported in the literature. Trustworthiness of the findings was assessed by external validation and investigator triangulation throughout data analysis. There were no Aboriginal or Torres Strait Islander participants; therefore, their perspectives have not been represented signifying a gap in knowledge for this priority population. The inclusion criteria of participants being 3 years or less since cancer diagnosis or recurrence were chosen to maximise recall and recency of cancer treatment experience; however, it is recognised this limitation may have excluded participants with experience of clinical trial participation. Due to the nature of qualitative research, the capacity to generalise these results to a wider population of people with ovarian cancer represents a limitation of this study, and as such, further investigation in a wider population group is recommended.

## Conclusions

The perspectives of Australians with lived experience of ovarian cancer indicated a need for more information on clinical trials to improve awareness and address barriers faced in accessing information and participating in clinical trials. These reflections were contextualised through varied emotional impacts and motivation to help others. These qualitative data provide crucial insights for the development of a national survey to further investigate these issues and ensure investment in newly developed information resources is tailored to meet the needs of people with ovarian cancer in Australia.

## Supplementary Information

Below is the link to the electronic supplementary material.

Supplementary file1 (PDF 292 KB)

## Data Availability

No datasets were generated or analysed during the current study.
